# Hybrid Radar Emitter Recognition Based on Rough *k*-Means Classifier and Relevance Vector Machine

**DOI:** 10.3390/s130100848

**Published:** 2013-01-11

**Authors:** Zhutian Yang, Zhilu Wu, Zhendong Yin, Taifan Quan, Hongjian Sun

**Affiliations:** 1 School of Electronics and Information Technology, Harbin Institute of Technology, Harbin 150001, China; E-Mails: deanzty@gmail.com (Z.Y.); wuzhilu@hit.edu.cn (Z.W.); quantf@hit.edu.cn (T.Q.); 2 Department of Electronic Engineering, King's College London, Strand, London, WC2R 2LS, UK; E-Mail: hongjian.sun@kcl.ac.uk

**Keywords:** hybrid recognition, rough boundary, uncertain boundary, computational complexity

## Abstract

Due to the increasing complexity of electromagnetic signals, there exists a significant challenge for recognizing radar emitter signals. In this paper, a hybrid recognition approach is presented that classifies radar emitter signals by exploiting the different separability of samples. The proposed approach comprises two steps, namely the primary signal recognition and the advanced signal recognition. In the former step, a novel rough *k*-means classifier, which comprises three regions, *i.e.*, certain area, rough area and uncertain area, is proposed to cluster the samples of radar emitter signals. In the latter step, the samples within the rough boundary are used to train the relevance vector machine (RVM). Then RVM is used to recognize the samples in the uncertain area; therefore, the classification accuracy is improved. Simulation results show that, for recognizing radar emitter signals, the proposed hybrid recognition approach is more accurate, and presents lower computational complexity than traditional approaches.

## 1. Introduction

Radar emitter recognition is a critical function in radar electronic support systems for determining the type of radar emitter [1]. Emitter classification based on a collection of received radar signals is a subject of wide interest in both civil and military applications. For example, in battlefield surveillance applications, radar emitter classification provides an important means to detect targets employing radars, especially those from hostile forces. In civilian applications, the technology can be used to detect and identify navigation radars deployed on ships and cars used for criminal activities [2]. This technology can be also applied in navigation radars for detecting ships and estimating their sizes [3], focusing on future classification stages [4].

The recent proliferation and complexity of electromagnetic signals encountered in modern environments greatly complicates the recognition of radar emitter signals [1]. Traditional recognition methods are becoming inefficient against this emerging issue [5]. Many new radar emitter recognition methods were proposed, e.g., intra-pulse feature analysis [6], stochastic context-free grammar analysis [1], and artificial intelligence analysis [7–11]. In particular, the artificial intelligence analysis approach has attracted much attention. Artificial intelligence techniques have been also successfully applied when working with radars for other purposes, such as clutter reduction stages [12], in target detection stages [13,14] and in target tracking stages [15]. Among the artificial intelligence approaches, the neural network and the support vector machine (SVM) are widely used for radar emitter recognition. In [8], Zhang *et al.* proposed a method based on the rough sets theory and radial basis function (RBF) neural network. Yin *et al.* proposed a radar emitter recognition method using the single parameter dynamic search neural network [9]. However, the prediction accuracy of the neural network approaches is not high and the application of neural networks requires large training sets, which may be infeasible in practice. Compared to the neural network, the SVM yields higher prediction accuracy while requiring less training samples. Ren *et al.*[2] proposed a recognition method using fuzzy C-means clustering SVM. Lin *et al.* proposed to recognize radar emitter signals using the probabilistic SVM [10] and multiple SVM classifiers [11]. These proposed SVM approaches can improve the accuracy of recognition. Unfortunately, the computational complexity of SVM increases rapidly with the increasing number of training samples, so the development of classification methods with high accuracy and low computational complexity is becoming a focus of research. Recently, a general Bayesian framework for obtaining sparse solutions to regression and classification tasks named relevance vector machine (RVM) was proposed. RVM is attracting more and more attention in many fields, including radar signal analysis [16,17].

Classifiers can be categorized into linear classifiers and nonlinear classifiers. A linear classifier can classify linear separable samples, but cannot classify linearly inseparable samples efficiently. A nonlinear classifier can classify linearly inseparable samples; nevertheless it usually has a more complex structure than a linear classifier and the computational complexity of the nonlinear classifier will be increased when processing linearly separable samples. In practice, the radar emitter signals consist of both linearly separable samples and linearly inseparable samples, which makes classification challenging, so in an ideal case, linearly separable samples should are classified by linear classifiers, while only these linearly inseparable samples are classified by the nonlinear classifier. However in the traditional recognition approach, only one classifier is used; thus, it is difficult to classify all radar emitter signal samples.

In this paper, a hybrid recognition method based on the rough *k*-means theory and the RVM is proposed. To deal with the drawback of the traditional recognition approaches, we apply two classifiers to recognize linearly separable samples and linearly inseparable samples, respectively. Samples are firstly recognized by the rough *k*-means classifier, while linearly inseparable samples are picked up and further recognized by using RVM in the advanced recognition. This approach recognizes radar emitter signals accurately and has a lower computational complexity.

The rest of the paper is organized as follows. In Section 2, a novel radar emitter recognition model is proposed. In Section 3, the primary recognition is introduced. In Section 4, the advanced recognition is introduced. In Section 5, the computational complexity of this approach is analyzed. The performance of the proposed approach is analyzed in Section 6, and conclusions are given in Section 7.

## 2. Radar Emitter Recognition System

A combination of multiple classifiers is a powerful solution for difficult pattern recognition problems. Thinking about the structure, a combined classifier can be divided into serial and concurrent. A serial combined classifier usually has a simple structure and is easy to establish. In serial combined classifiers, the latter classifier makes the samples rejected by the former its training samples. Thus in designing it, the key is choosing the complementary classifiers and determining the rejected samples.

In this section, a hybrid radar emitter recognition approach that consists of a rough *k*-means classifier in the primary recognition and a RVM classifier in the advanced recognition is proposed. This approach is based on the fact that in the *k*-means clustering, the linearly inseparable samples are mostly at the margins of clusters, which makes it difficult to determine which cluster they belong to. To solve this problem, in our approach a linear classifier and a nonlinear classifier are applied to form a hybrid recognition method. In the proposed approach, the rough *k*-means classifier, which is linear, is applied as the primary recognition. It can classify linearly separable samples and pick up those linearly inseparable samples to be classified in the advanced recognition.

**Figure 1.**
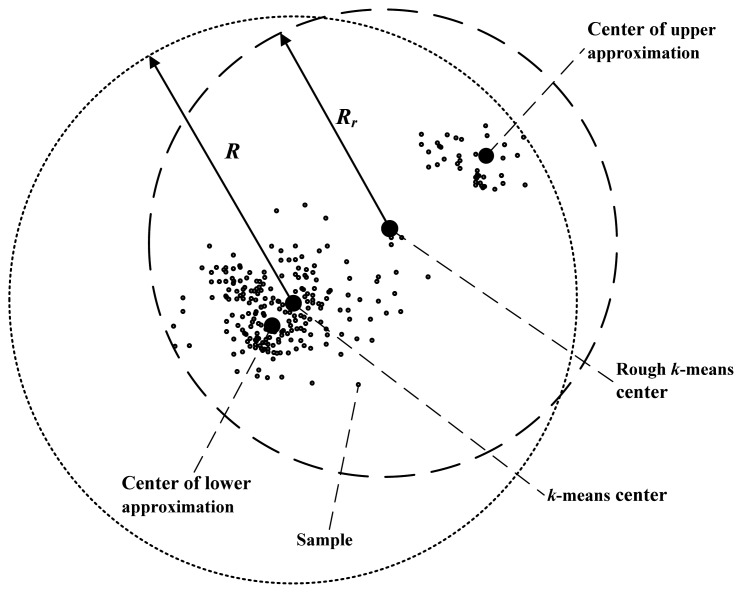
Regions of the rough *k*-means classifier: the certain, the rough and the uncertain area. Linearly separable samples are usually near to the center, while linearly inseparable samples are usually far from the center.

In the rough *k*-means algorithm, there are two areas in a cluster, *i.e.*, certain area and rough area. But in the rough *k*-means classifier proposed in this paper, there exist three areas, *i.e.*, certain area, rough area and uncertain area. For example, in two dimensions, a cluster is depicted in Figure 1.

Training samples are clustered first. At the edge of the cluster, there is an empty area between the borderline and the midcourt line of the two cluster centers. We name this area as the uncertain area. In clustering, there is no sample in the uncertain area. When the clustering is completed, these clusters will be used as the minimum distance classifier. When unknown samples are classified, samples are distributed into the nearest cluster. However linearly inseparable samples are usually far from cluster centers and out of the cluster probably, *i.e.*, in the uncertain area. Thus after distributed into their nearest clusters, the unknown samples in the uncertain area will be recognized by the advanced recognition using a nonlinear classifier. For those unknown samples in the certain area and rough area, the primary recognition outputs final results.

**Figure 2.**
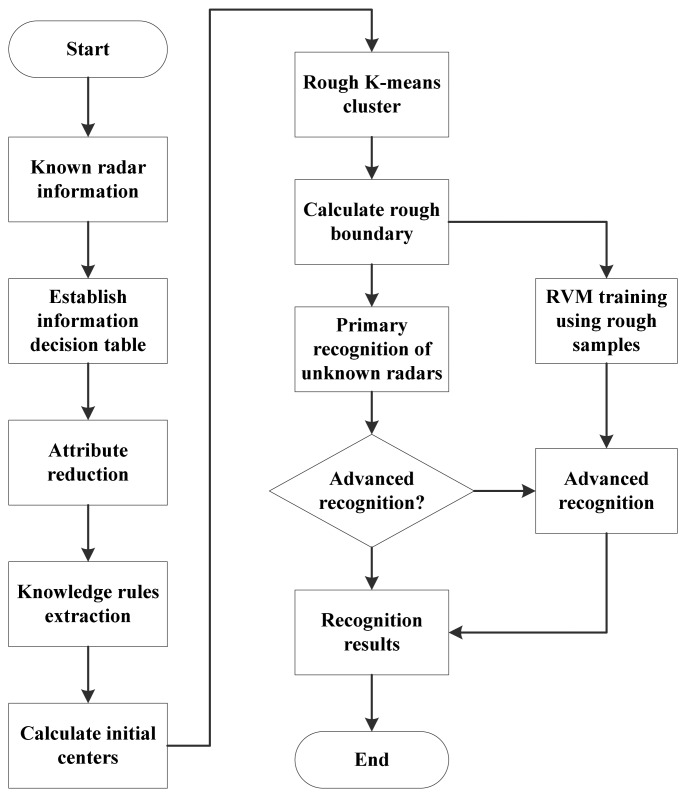
Flow chart of the hybrid radar emitter recognition approach proposed in this paper. First of all, samples are recognized by the primary recognition, which can classify linearly separable samples and pick up those linearly inseparable samples to be classified in the advanced recognition using relevance vector machine.

After sorting and feature extraction, radar emitter signals are described by pulses describing words. Radar emitter recognitions are based on these pulses describing words. The process of the hybrid radar emitter recognition approach is shown in Figure 2.

Based on the pulses describing words, we can obtain an information sheet of radar emitter signals. By using rough sets theory, the classification rules are extracted. These classification rules are the basis of the initial centers of the rough *k*-means classifier. More specifically, they determine the initial centers and the number of clusters. After that, the known radar emitter signal samples are clustered by the rough *k*-means while the rough *k*-means classifier in the primary recognition is built, as described in the next section. The samples in the margin of a cluster are affected easily by noises and even out of the cluster boundary, which will cause confusions in recognition of unknown samples. Thus, the samples in the margin of a cluster are picked up to be used as the training data for the RVM in the advanced recognition. In recognition, the unknown samples to be classified are recognized firstly by the rough *k*-means classifier. The uncertain sample set, which is rejected by the primary recognition, is classified by the RVM in the advanced recognition. In the advanced recognition, RVM will recognize these unknown samples based on the training samples, *i.e.*, the samples in the rough areas. More specifically, the samples which are the rough samples affected by the noise, will be recognized. And other samples will be rejected by the advanced recognition.

Based on the process of the recognition approach described above, the accuracy of the hybrid recognition is a superposition of two parts, *i.e.*, the accuracy of the primary recognition and the accuracy of the advanced recognition. The samples that the primary recognition rejects are classified by the advanced recognition. So the estimate of recognition accuracy can be given by:

(1) $$A_{\text{total}}=A_{\text{primary}}+R_{\text{primary}}\times A_{\text{advanced}}$$

where *A_total_*, *A_primary_*, *A_advanced_*, and *R_primary_* denote the accuracy of the hybrid recognition, the accuracy of the primary recognition, the accuracy of the advanced recognition, and the reject rate of the primary classifier, respectively.

## 3. Primary Recognition Based on Improved Rough *k*-means

As mentioned above, a classifier based on the rough *k*-means is proposed as the primary recognition. Rough *k*-means is a generation of *k*-means algorithm, which is one of the most popular iterative descent clustering algorithms [18]. The basic idea of *k*-means algorithm is to make the samples have high similarity in a class, and low similarity among classes. However *k*-means clustering algorithm has the following problems:

1. The number of clusters in the algorithm must be given before clustering.
2. The *k*-means algorithm is very sensitive to the initial center selection and can easily end up with a local minimum solution.
3. The *k* -means algorithm is also sensitive to isolated points.

To overcome the problem of isolated points, Pawan and West proposed the rough *k*-means algorithm [19]. The rough *k*-means can solve the problems of nondeterminacy in clustering and reduce the effect of isolated samples efficiently, but it still requires initial centers and the number of clusters as priors. In this paper, we propose to determine the number and initial centers of clusters based on rough sets theory.

In rough sets theory, an information system can be expressed by a four-parameters group [20]: *S* = {*U*, *R*, *V*, *f*}. *U* is a finite and non-empty set of objects called the universe, and *R* = *C* ∪ *D* is a finite set of attributes, where *C* denotes the condition attributes and *D* denotes the decision attributes. *V* = ∪*v_r_*, (*r* ∈ *R*) is the domain of the attributes, where *v_r_* denotes a set of values that the attribute *r* may take. *f: U* × *R* → *V* is an information function. The equivalence relation *R* partitions the universe *U* into subsets. Such a partition of the universe is denoted by *U*/*R* = *E*_1_, *E*_2_,…, *E_n_*, where *E_i_* is an equivalence class of *R*. If two elements *u*, *v* ∈ *U* belong to the same equivalence class E ⊆ *U*/*R*, *u* and *v* are indistinguishable, denoted by *ind*(*R*). If *ind*(*R*) = *ind*(*R*–*r*), *r* is unnecessary in *R*. Otherwise, *r* is necessary in *R*.

Since it is not possible to differentiate the elements within the same equivalence class, one may not obtain a precise representation for a set *X* ⊆ *U*. The set X, which can be expressed by combining sets of some R basis categories, is called set defined, and the others are rough sets. Rough sets can be defined by upper approximation and lower approximation. The elements in the lower bound of *X* definitely belong to *X*, and elements in the upper bound of *X* belong to *X* possibly. The upper approximation and lower approximation of the rough set *R* can be defined as follows [20]:

(2) $$\underline{R}(X)=\cup \{Y\in \frac{U}{R:Y\subseteq X}\}$$

(3) $$\bar{R}(X)=\cup \{Y\in \frac{U}{R:Y\cap X\neq ⊘}\}$$

where *Ṟ*(*X*) represents the set that can be merged into *X* positively, and *R̄*(*X*) represents the set that is merged into *X* possibly.

In the radar emitter recognition, suppose *Q* is the condition attribute, namely, the pulse describing words for classification, *P* is the decision attribute, namely, the type of radar emitter, and the *U* is the set of radar emitter samples. The information systems decided by them are *U*/*P* = {[*x*]*_P_*|*x* ∈ *U*} and *U*/*Q* = {[*y*]*_P_*|*y* ∈ *U*}. If for any [*x*]*_P_* ∈ (*U*/*P*):

(4) $$\bar{Q}\left({\left[x\right]}_{P}\right)=Q\left({\left[x\right]}_{P}\right)={\left[x\right]}_{P}$$

then *P* is dependent on *Q* completely, that is to say when disquisitive radar emitter sample is some characteristic of *Q*, it must be some characteristic of *P. P* and *Q* are of definite relationship. Otherwise, *P* and *Q* are of uncertain relationship. The dependent extent of knowledge *P* to knowledge *Q* is defined by:

(5) $$\gamma_{Q}=POS_{P}(Q)/|U|$$

where *POS_P_*(*Q*) = ∪*Q̱*(*x*) and 0 ≤ *γ_Q_* ≤ 1. The value of *γ_Q_* reflects the dependent degree of *P* to *Q. γ_Q_* = 1 shows *P* is dependent on *Q* completely; *γ_Q_* close to 1 shows *P* is dependent on *Q* highly; *γ_Q_* = 0 shows *P* is independent of *Q* and the condition attribute *Q* is redundancy for classification. Due to the limitation of length, rough sets theory is introduced briefly here. And the details of rough sets are introduced in reference [20].

After discretization and attribute reduction, the classification rules are extracted. Using this approach, the initial centers are computed based on the classification rules of rough sets. The process can be described as follows:

1. Classification rules are obtained based on the rough sets theory.
2. The mean value of every class is obtained.
3. The clustering number equals to the number of rules and define the mean values as the initial clustering centers:
  (6) $$t_{p}=\frac{\sum_{x\in X_{p}}x}{\text{card}(X_{p})}$$
  where *X_p_* denotes the set of samples in the classification rule *p* of the rough sets theory.

In rough *k*-means algorithm upper approximation and lower approximation are introduced. The improved cluster center is given by [19]:

(7) $$C_{j}=\{\begin{array}{cc} \omega_{\text{lower}}\times \frac{\sum_{v\in \underline{A}(x)}v_{j}}{\left|\underline{A}(x)\right|}+\omega_{\text{upper}}\times \frac{\sum_{v\in (\bar{A}(x)-\underline{A}(x))}v_{j}}{\left|\bar{A}(x)-\underline{A}(x)\right|} & \text{if}\bar{A}(x)-\underline{A}(x)\neq ⊘\\ \omega_{\text{lower}}\times \frac{\sum_{v\in \underline{A}(x)}v_{j}}{\left|\underline{A}(x)\right|} & \text{otherwise} \end{array}$$

where the parameters *ω_lower_* and *ω_upper_* are lower and upper subject degrees of *x* relative to the clustering center. For each object vector *v*, *d*(*x*, *t_i_*) (1 ≤ *i*≤ *I*) denotes the distance between the center of cluster *t_i_* and the sample. The lower and upper subject degrees of *x* relative to its cluster is based on the value of *d*(*x*,*t_i_*)−*d_min_*(*x*), where *d_min_*(*x*) = min*_i_*_∈[1_, *_I_*_]_*d*(*x*,*t_i_*). If the value of *d*(*x*,*t_i_*) − *d_min_*(*x*) ≥ *λ*, the sample *x* is subject to the lower approximation of its cluster, where *λ* denotes the threshold for determining upper and lower approximation. Otherwise, *x* will be subject to the upper approximation. The comparative degree can be determined by the number of elements in the lower approximation set and the upper approximation set, as follows:

(8) $$\frac{\omega_{\text{lower}}(i)}{\omega_{\text{upper}}(i)}=\frac{\left|\bar{A}(X_{i})\right|}{\left|\underline{A}(X_{i})\right|},(\underline{A}(X_{i})\neq ⊘)$$

(9) $$\omega_{\text{lower}}(i)+\omega_{\text{upper}}(i)=1$$

In Equation (7), the parameter *λ* determines the lower and upper subject degree of *X_k_* relative to some clustering. If the threshold *λ* is too large, the low approximation set will be empty, while if the threshold *λ* is too small, the boundary area will be powerless. The threshold *λ* can be determined by:

1. Compute the Euler distance of every object to *K* class clustering centers and distance matrix *D*(*i*, *j*)
2. Compute the minimum value *d_min_*(*i*) in every row of matrix *D*(*i*, *j*)
3. Compute distance between every object and other class center *d_i_* and *d_t_*(*i*, *j*)=*d*(*i*)-*d_min_*(*i*)
4. Obtain the minimum value *d_s_*(*i*) (except zero) in every row
5. *λ* is obtained from the minimum value *d_s_*(*i*)

In the training process of the rough *k*-mean classifier, we need calculate the cluster center; rough boundary *R_ro_* and uncertain boundary *R_un_* in every cluster. After clustering, the center of a cluster and the farthest sample from the center of the cluster are determined. The area between rough boundary and uncertain boundary (*R_ro_* < *d_x_*< *R_un_*) is defined as rough area, where *d_x_* denotes the distance from a sample to the center. In the training, if a training sample is in the rough area, it will be used to train the RVM in the advanced recognition. The uncertain boundary threshold *R_un_* is defined by:

(10) $$R_{un}=\max(d_{x})$$

where max(*d_x_*) is the distance from the farthest sample to the center. The rough radius *R_ro_* can be defined by:

(11) $$R_{ro}=\delta R_{un}$$

and the scale factor *δ* ∈ [0.7,0.9] generally. In this paper, *δ* = 0.8.

In a cluster, the area beyond uncertain boundary (*d_x_* > *R_un_*) is the uncertain area. When unknown samples are recognized, they will be distributed into the nearest cluster. If *d_x_* > *R_un_*, these samples will be further recognized by the advanced recognition. For other unknown samples, the result of the primary recognition will be final.

**Figure 3.**
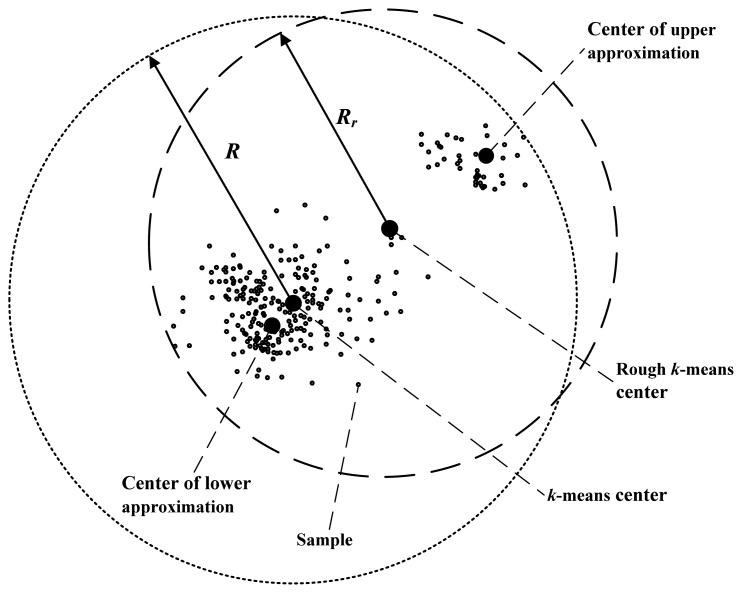
The radius of a cluster in rough *k*-means is shorter than that in *k*-means.

In addition, the accuracy of primary recognition is relevant with the radii of clusters. Rough *k*-means clustering can lessen the radii of clusters effectively. Comparison of radii of the rough *k*-means cluster and the *k*-means cluster is shown in Figure 3.

As shown in Figure 3, the radius of the *k*-means cluster is the distance from the cluster center to the farthest isolated sample. In the rough *k*-means, the cluster center is the average of the lower approximation center and the upper approximation center. The upper approximation center is near to the farthest sample, so the cluster radius of rough *k*-means *R_r_* is less than the *k*-means radius *R*, obviously. As the radius is shortened, when unknown samples are recognized, the probability that an uncertain sample is recognized as a certain sample is reduced. Therefore, the accuracy of the primary recognition is increased.

## 4. The Advanced Recognition Using RVM

The relevance vector machine (RVM), a sparse Bayesian modeling approach, is proposed by Tipping [21], which enables sparse classification by linearly-weighting a small number of fixed basis functions from a large dictionary of potential candidates. And a significant advantage to support vector machine is that the kernel function of RVM avoids satisfying Mercer's condition [22–24].

In classification, the output function *y*(*x*) is defined by:

(12) $$y\left(x,\omega \right)=\sigma (\omega^{\text{T}}\phi \left(x\right))$$

where *σ*(*z*) = 1/(1+e^−^*^z^*) and **ω** denotes the weight matrix.

Suppose **ω** is to a Gauss conditional probability, with the 0 expectation and variance $a_{i}^{-1}$. For two classes classification, the likelihood function is defined by:

(13) $$\text{P}\left(t|\omega \right)=\prod_{n-1}^{N}\sigma {\left\{y\left(x_{n},\omega \right)\right\}}^{t_{n}}{\left[1-\sigma \left\{y\left(x_{n},\omega \right)\right\}\right]}^{1-t_{n}}$$

where *t_n_* ∈ (0,1) denote the target value.

Seeking the maximum posterior probability estimation is equivalent to seeking the mode point of the Gaussian function, namely, *μ_MP_*.

Due to:

(14) $$\text{P}\left(\omega |t,\alpha \right)=\frac{\text{P}\left(t|\alpha \right)\text{P}\left(\omega |\alpha \right)}{\text{P}\left(t|\alpha \right)}$$

the maximum posterior probability estimation according to **ω** is equivalent to maximize:

(15) $$\begin{array}{l} \log\left\{\text{P}\left(\omega |t,\alpha \right)\right\}=\text{log}\left\{\text{P}\left(t|\omega \right)\right\}+\log\left\{\text{P}\left(\omega |\alpha \right)\right\}-\text{log}\left\{\text{P}\left(t|\alpha \right)\right\}\\ =\sum_{n=1}^{N}\left[t_{n}\log y_{n}+\left(1-t_{n}\right)\log\left(-y_{n}\right)\right]-\frac{1}{2}\omega^{\text{T}}A\omega +C \end{array}$$

where *y_n_* = *σ*{*y*(*x_n_*,**ω**)}, *C* denotes a constant. Similarly, the marginal likelihood function can be given by:

(16) $$\text{P}\left(\omega |t,\alpha \right)=\int \text{P}\left(t|\omega \right)\text{P}\left(\omega |\alpha \right)d\omega \text{P}\left(t|\omega_{MP}\right)\text{P}\left(\omega_{MP}|\alpha \right){\left(2\pi \right)}^{M/2}{|\sum |}^{1/2}$$

Suppose **t̂** = **Φ_ωMP_** + **B**^−1^(**t** − **y**), the approximation of the Gaussian posterior distribution, *i.e.*, *μ_MP_* = ΣΦ*^T^***Bt̂**, with the variance Σ = (Φ*^T^***B**Φ + **A**) ^−1^. The logarithm of the approximate marginal likelihood function is given by:

(17) $$\log\text{p}\left(t|\alpha \right)=-\frac{1}{2}\left\{N\log\left(2\pi \right)+\log\left|C\right|+{\hat{t}}^{T}C^{-1}\hat{t}\right\}$$

where **C** = **B** + Φ**A**^−1^Φ*^T^*

A fast marginal likelihood maximisation for sparse Bayesian models is proposed in reference [21], which can reduce the learning time of RVM effectively. To simplify forthcoming expressions, it is defined that:

(18) $$s_{i}=\phi_{i}^{T}C_{-i}^{-1}\phi_{i}$$

(19) $$q_{i}=\phi_{i}^{T}C_{-i}^{-1}t$$

It is showed that Equation (16) has a unique maximum with respect to *α_i_*:

(20) $$\alpha_{i}=\frac{{s_{i}}^{2}}{q_{i}^{2}-s_{i}},\text{if}\;{q_{i}}^{2}>s_{i},$$

(21) $$\alpha_{i}=\infty ,\text{if}\;{q_{i}}^{2}\leq s_{i}$$

The proposed marginal likelihood maximization algorithm is as follows:

1. Initialize with a single basis vector *φ_i_*, setting, from Equation (20):
  (22) $$\alpha_{i}=\frac{{\left\|\phi_{i}\right\|}^{2}}{{\left\|{\phi_{i}}^{\text{T}}\right\|}^{2}/{\left\|\phi_{i}\right\|}^{2}-\sigma^{2}}.$$
2. Compute Σ and μ (which are scalars initially), along with initial values of sm and qm for all M bases *ϕ*_m_.
3. Select a candidate basis vector *φ_i_* from the set of all M.
4. Compute $\theta_{i}=q_{i}^{-2}-S_{i}$.
5. If *θ*_i_ > 0, *α*_i_ < ∞, re-estimate *α*_i_.
6. If *θ*_i_ > 0, *α*_i_ = ∞, add *φ_i_* to the model with updated *α*_i_.
7. If *θ*_i_ ≤ 0, *α*_i_ < ∞, delete *φ_i_* from the model and set *α*_i_ = ∞.
8. Recompute and update Σ, *μ*, *s_m_* and *q_m_*, where, $s_{m}=\frac{\alpha_{m}S_{m}}{\alpha_{m}-S_{m}}$, $q_{m}=\frac{\alpha_{m}Q_{m}}{\alpha_{m}-S_{m}}$, *S_m_* = *φ_m_*^T^**B***φ_m_-φ_m_*^T^**B**ΦΣΦ^T^**B***φ_m_* and *Q_m_* = *φ_m_*^T^**Bt̂**-*φ_m_*^T^**B**ΦΣΦ^T^**Bt̂**.
9. If converged, terminate the iteration, otherwise go to 3.

The fast marginal likelihood maximisation for sparse Bayesian models is stated in details in [21,22].

## 5. Computational Complexity Analysis

The computational complexity of the approach proposed in this paper consists of two parts, namely the computational complexity of the primary recognition and the computational complexity of the advanced recognition.

In the training of the primary recognition, samples are clustered using rough *k*-means. The computational complexity of the rough *k*-means is *O*(*dmt*), where *d*, *m* and *t* denote the dimension of samples, the number of training samples and the iterations, respectively. In this paper, the optimal initial centers are determined by analyzing the knowledge rule of the training sample set based on rough set theory, instead of iteration. Thus, the computational complexity of the primary recognition is *O*(*dm*).

The RVM is used as the advanced recognition in our approach. The computational complexity of RVM has nothing with the dimension of samples, but is related with the number of samples. The computational complexity of RVM training is discussed with respect to the complexity of the quadratic programming. RVM training has a computational complexity less than *O*(*m*′^3^), where *m*′ denotes the number of training samples for RVM in the advanced recognition [22].

In conclusion, the computational complexity of our hybrid recognition is *O*(*dm*) + *O*(*m*′^3^). In general, *O*(*dm*) ≪ *O*(*m*′3). Therefore, the computational complexity of the hybrid recognition training is regard as *O*(*m*′^3^). In actual practice, *m*′ is not larger than the training sample number, *i.e.*, *m*[22]. *m*′ will be lessened with the reduction of *m*. In the primary recognition, training samples are differentiated and only a part of samples, namely uncertain samples, are used for RVM training. Therefore, the proposed approach can present lower computational cost than RVM.

## 6. Results and Discussion

The validity and efficiency of the proposed approach is proved by simulations. In the first simulation, radar emitter signals are recognized. The pulse describing words of the radar emitter signal include a radio frequency (RF), a pulse repetition frequency (PRF), antenna rotation rate (ARR) and a pulse width (PW). The type of radar emitter is the recognition result. Two hundred and seventy groups of data are generated on above original radar information for training. And the recognition accurate is calculated averaged over 200 random generations of the data set.

Another simulation is adopted to test the generalization of the hybrid recognition with the Iris data set. The Iris data set contains 150 patterns belonging to three classes. There are 50 exemples for each class and each input is a four-dimensional real vector [25]. The recognition accuracy and computational complexity are compared with SVM and RVM. This simulation consists of two parts. In the first part, all 150 samples are used in training, while all of 150 samples are used to test the training accuracy. In the second part, 60 random samples are used to train classifiers and other 90 samples are used to test the generalization. Simulations are run on a personal computer, equipped with a Pentium (R) Dual 2.2 GHz processor and 2G RAM.

### 6.1. Results of Experiment 1: Classification of the Radar Emitter Signals

An information sheet of radar emitter signals is built, which is shown as Table 1. Nine known radar emitter signals are applied to test the proposed approach.

Training and test samples are random generations of the data set shown in Table 1. Data in the information table should be changed into discrete values, because continuous values cannot be processed by the rough sets theory. There are many methods for data discretization and here the equivalent width method [20] is applied in this paper. In our paper, attributes are divided into three intervals. The attribute values in the same interval have the same discrete value. In discretization, samples with the same discrete condition attribute values are merged into a discrete sample in Table 2 (one row). A, B, C and d denote the attributes RF, PRF, PW and type, respectively.

After that, the dependent extent of radar type to each attribute is computed using Equation (3). The degrees of attribute importance can be calculated, *i.e.*, *σ_D_*(A) = 1/2, *σ_D_*(B) = 3/8 and *σ_D_*(C) = 0. As the dependent extent of radar type to the attribute C (PW) is 0, the attribute C is unnecessary for classification and removed. After redundancy attributes and repeated samples are removed, the knowledge rules are obtained. Table 3 shows these rules, where - denotes the arbitrary value.

As shown in Table 3, six rules are extracted, which means that 270 samples from three types of radar emitter can be classified into six subclasses. Based on these knowledge rules, initial clustering centers are obtained using Equation (6). The known radar emitter samples are clustered by using the rough *k*-means on these initial cluster centers. The cluster centers, rough boundary and uncertain boundary of the primary recognition are computed. The information of clusters is shown in Table 4. The rough *k*-means classifier has been built and rough samples are picked up. RVM in the advanced recognition are trained using these rough samples.

In recognition of unknown samples, some important parameters are computed in the simulation. The accuracy, error and reject rate of the primary recognition are 86%, 2.5%, 11.5%, respectively. The accuracy of advanced recognition is 93.1%. Thus, the estimate of accuracy can be computed as: *A_total_* = 86% + 11.5% × 91.3% = 96.5%.

The proposed method is compared with the RBF-SVM, the probabilistic SVM radar recognition approach studied by Lin *et al.* in [10] and RVM studied by Tipping [22]. The training accuracy, training time and recognition accuracy are shown in Table 5.

As shown in Table 5, the four approaches achieve high training accuracies. The training accuracy of the approach proposed in this paper achieves 99.5%, which indicates this approach has good fitting capacity to the training samples. The accuracy of the hybrid recognition proposed in this paper is 96.5%, which is higher than existing methods, *i.e.*, 94.0%, 93.5% and 94.0%. The accuracy of the hybrid recognition from simulation experiments accords with the theoretical value, *i.e.*, 96.5%. Moreover, SVM approaches need less train time than RVM. The training time of the proposed hybrid recognition is least in these four approaches, *i.e.*, 2.1 s. The hybrid recognition has a faster training because of lower computational complexity. And the training computational complexities of approaches will be analyzed behind.

### 6.2. Results of Experiment 2: Classification of the Iris Data Set

In the first part, all 150 of the samples are used for training and testing. In this simulation, the training accuracy of the hybrid recognition is tested. In addition, the accuracy of recognition and computational complexity of the hybrid recognition is compared with those of SVM and RVM. The results are as shown in Table 6.

From Table 6, we can know the proposed approach has a higher training accuracy than SVM and RVM. In the first part of this experiment, all 150 samples are used to train and test these methods. The hybrid recognition proposed in this paper has a high training accuracy, *i.e.*, 99.33%, which is higher than those of other approaches, *i.e.*, 98.00% and 98.67%.

In the second part, 60 random samples from Iris are used to train classifiers and other 90 samples are used for test to test the generalization. The accuracy of recognition and computational complexity of the hybrid recognition is compared with those of SVM and RVM. The results are as shown in Table 7.

The recognition accuracy of the proposed approach is 96.67%, which is higher than those of other approaches. It is indicated that the hybrid recognition has not only a high training accuracy but also a good generalization.

In addition, let's compare the training computational complexities of SVM, RVM and the proposed approach. The computational complexity of SVM is *O*(*m*^3^). The computational complexity of RVM is *O*(*m*^3^). The computational complexity of the proposed approach is *O*(*m*′^3^), where *m*′ denotes the number of training samples for the RVM in the advanced recognition of the hybrid recognition. When 150 samples are used as training samples, all of them are used to train the SVM and RVM, namely, *m* = 150. The time complexities of the classical SVM and RVM are *O*(150^3^). In our approach, training samples are clustered in the primary recognition, and only the rough samples are used to train the RVM in the advanced recognition. More specifically, there are 71 training samples for the RVM in the advanced recognition, *i.e.*, *m*′ = 71, so it's computational complexity is *O*(71^3^). Similarly, when 60 samples are used as training samples, all of these samples are used to train SVM and RVM, while 36 training samples are picked up for the RVM in the advanced recognition of the hybrid recognition, *i.e.*, *m* = 60 and *m*′ = 36. So in the second part, the computational complexity of SVM and RVM is *O*(60^3^), while the computational complexity of the proposed approach is *O*(36^3^). From the comparison above, we can know that the computational complexity of the hybrid recognition is obviously lower than those of RVM and SVM.

Theoretically, lower computational complexity leads to less computational time. The actual calculation time for each algorithm is tested and the result is shown in Table 7. The training calculation time of the proposed hybrid recognition is obviously less than SVM and RVM. Compared with SVM, a distinct advantage of RVM is the sparse structure. Although the computational complexity of RVM training is close to the SVM's, the discrimination process of RVM is more succinct and rapid than the SVM's. The proposed hybrid recognition approach inherits this superiority from RVM. The recognition time of the proposed approach is close to RVM and less than SVM.

## 7. Conclusions

In this paper, a hybrid recognition method has been proposed to recognize radar emitter signals. The hybrid classifier consists of a rough *k*-means classifier (linear classifier) and a RVM (nonlinear classifier). Based on the linear separability of the classifying sample, the sample is classified by the suitable classifier. Thus for the radar emitter sample set containing both linearly separable samples and linearly inseparable samples, the approach can achieve a higher accuracy.

A linear classifier based on the rough set and the rough *k*-means has been proposed, *i.e.*, the rough *k*-means classifier. The rough *k*-means clustering can reduce the radius of the clusters and increase the accuracy of the primary recognition. The initial centers for the rough *k*-means are computed based on the rough set, which can reduce the computational complexity of the rough *k*-means clustering. The rough *k*-means classifier can classify linear separable samples efficiently and pick up linearly inseparable samples. These linear inseparable samples are processed by the RVM in the advanced recognition. Therefore, the training samples for the RVM in the advanced recognition are reduced. Simulation results have shown that the proposed approach can achieve a higher accuracy, a lower computational complexity and less computation time, when compared with existing approaches.

The hybrid recognition approach in this paper is suitable for the classification of the radar emitter signal containing both linearly separable and linearly inseparable samples. However, for the situations where only linearly separable or linearly inseparable samples are included, the effectiveness of the hybrid approach will be not significant. We admit that our hybrid recognition approach is based on the fact that these linearly inseparable samples which reduce the accuracy of clustering are mostly at the edges of clusters. From Equation (1), we know that if the linearly inseparable sample appears frequently in the center region instead of the edge, the accuracy of recognition will be reduced. How to solve these problems is the focus of our future work.

## Figures and Tables

**Table 1.** Information of known radar emitter signals.

**No.**	**RF (MHz)**	**PRF (Hz)**	**PW (us)**	**Type**
1	8,799	1,500	0.1	1
2	8,847	750	0.5	1
3	8,755	620	0.5	2
4	8,890	580	0.5	2
5	8,875	585	0.5	2
6	8,804	750	0.1	1
7	8,850	1,500	0.5	1
8	9,460	1,300	0.25	3
9	9,436	1,600	0.15	3

**Table 2.** Continuous values are changed into discrete information by using the equivalent width method.

**No.**	**A**	**B**	**C**	**d**
1	1	3	1	1
2	2	2	3	1
3	1	2	3	2
4	2	1	3	2
5	2	1	3	2
6	2	2	1	1
7	2	3	3	1
8	3	3	2	3
9	3	3	1	3

**Table 3.** Classification rules are extracted based on rough sets theory. These rules are the basis of the choice of the initial centers in rough *k*-means cluster.

**No.**	**A**	**B**	**d**
1	-	1	2
2	1	2	2
3	2	2	1
4	1	3	1
5	2	3	1
6	3	3	3

**Table 4.** Centers, rough boundary radiuses and uncertain boundary radiuses of clusters.

**Cluster**	**Center**	***R****_ro_*	***R****_un_*
1	(8882.5, 582.5)	63	142
2	(8,755, 620)	70	128
3	(8,827, 750)	56	119
4	(8,799, 1,500)	37	41
5	(8,850, 1,500)	34	45
6	(9,448, 1,450)	398	607

**Table 5.** Training accuracy, training accuracy and recognition accuracy of radar emitter recognition approaches are compared.

**Recognition Approach**	**Training Accuracy**	**Training Time (s)**	**Recognition Accuracy**
RBF-SVM	99.5%	3.1	94.0%
PSVM	99.0%	3.4	93.5%
RVM	99.0%	4.6	94.0%
Method in this paper	99.5%	2.1	96.5%

**Table 6.** In the first part of experiment 2, the recognition accuracy of Iris data set and computational complexity are compared among three approaches.

**Approach**	**Accuracy**	***m* or *m***′	**Computational Complexity**	**Training Time (s)**
SVM	98.00%	150	*O*(150^3^)	0.9
RVM	98.67%	150	*O*(150^3^)	1.2
Hybrid recognition	99.33%	71	*O*(71^3^)	0.6

**Table 7.** In the second part of experiment 2, the recognition accuracy of Iris data set and computational complexity are compared among three approaches.

**Approach**	**Accuracy**	***m* or *m***′	**Computational Complexity**	**Training Time (s)**
SVM	93.33%	60	*O*(60^3^)	0.13
RVM	94.44%	60	*O*(60^3^)	0.14
Hybrid recognition	96.67%	36	*O*(36^3^)	0.04
